# Chronic Progressive Disseminated Histoplasmosis in a Mexican Cockfighter

**DOI:** 10.4269/ajtmh.14-0086

**Published:** 2015-01-07

**Authors:** René Agustín Flores-Franco, Antonio Gómez-Díaz, Antonio de Jesús Fernández-Alonso

**Affiliations:** Departments of Internal Medicine and Pathology, Regional General Hospital “Dr. Salvador Zubirán Anchondo,” Chihuahua, México

## Abstract

We present illustrative images from a Mexican 58-year-old man who had the occupation of cockfighting from childhood and presented with chronic progressive disseminated histoplasmosis with primarily cutaneous manifestations.

A 58-year-old man, a native of Tamazula, Durango in Mexico, complained of a 2-month history of odynophagia, hoarseness, non-productive cough, polyarthralgia, and weight loss. He denied fever and chills, but a few days after initial symptoms began, he presented erythematous papules and ulcers on the body trunk that subsequently spread to the extremities (Figure 1A and B). The patient had worked as a cockfight handler in traditional fairs during most of his life. No comorbidities were present, and a QuantiFERON-TB Gold Test in tube was negative. Computed tomography (CT) showed a stippled calcification on mediastinal lymph nodes (Figure 1C). It was after a second skin biopsy that the presence of yeasts consistent with histoplasmosis was finally seen (Figure 1D

**Figure 1.**
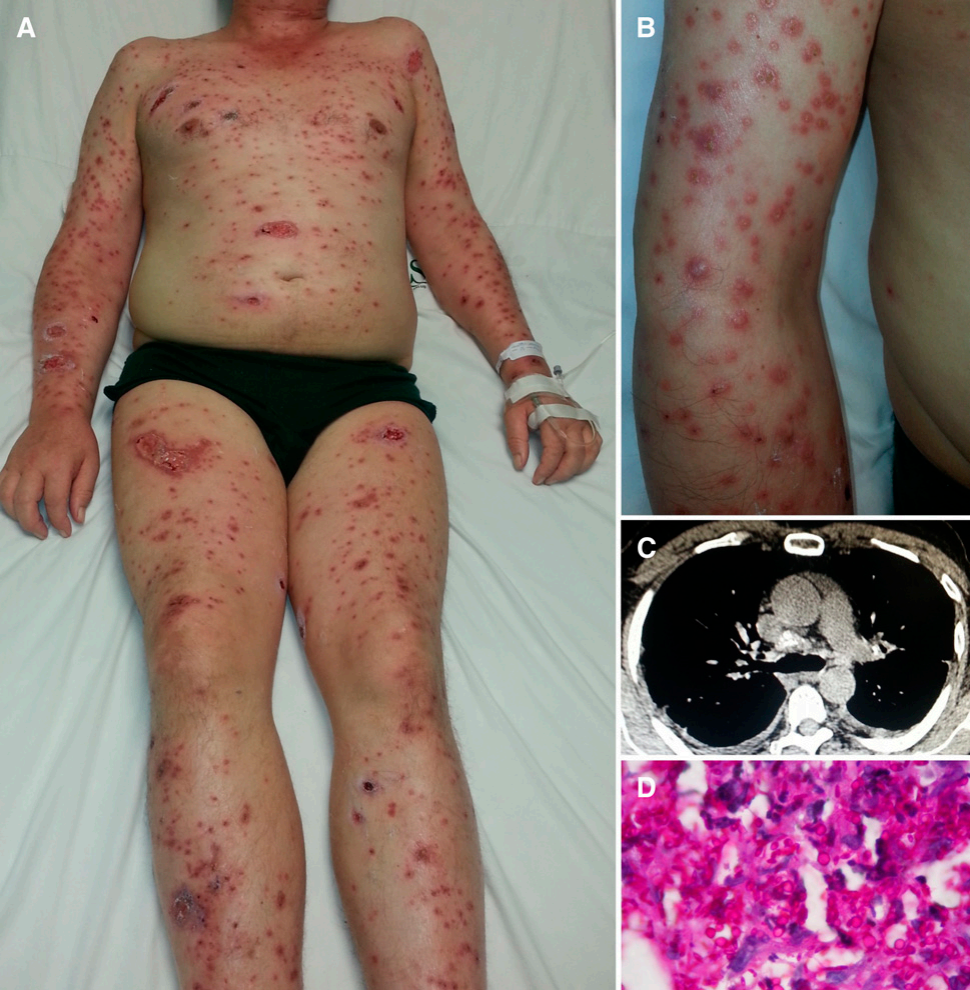
(**A**) Generalized nodule-ulcerative lesions characteristic of chronic progressive disseminated histoplasmosis. (**B**) On the right arm, there are multiple skin-colored umbilicated papules, hemorrhagic crusts, and ulcerated nodules. (**C**) CT of the mediastinum showing pretracheal and hilar calcified lymph nodes. (**D**) Photomicrograph of the pathologic specimen stained with periodic acid-Schiff shows numerous histiocytes and extracellular yeasts of *H. capsulatum* (magnification, ×400).

). He was started on itraconazole (400 mg/day), and 4 months later, his symptoms improved (Figure 2

**Figure 2.**
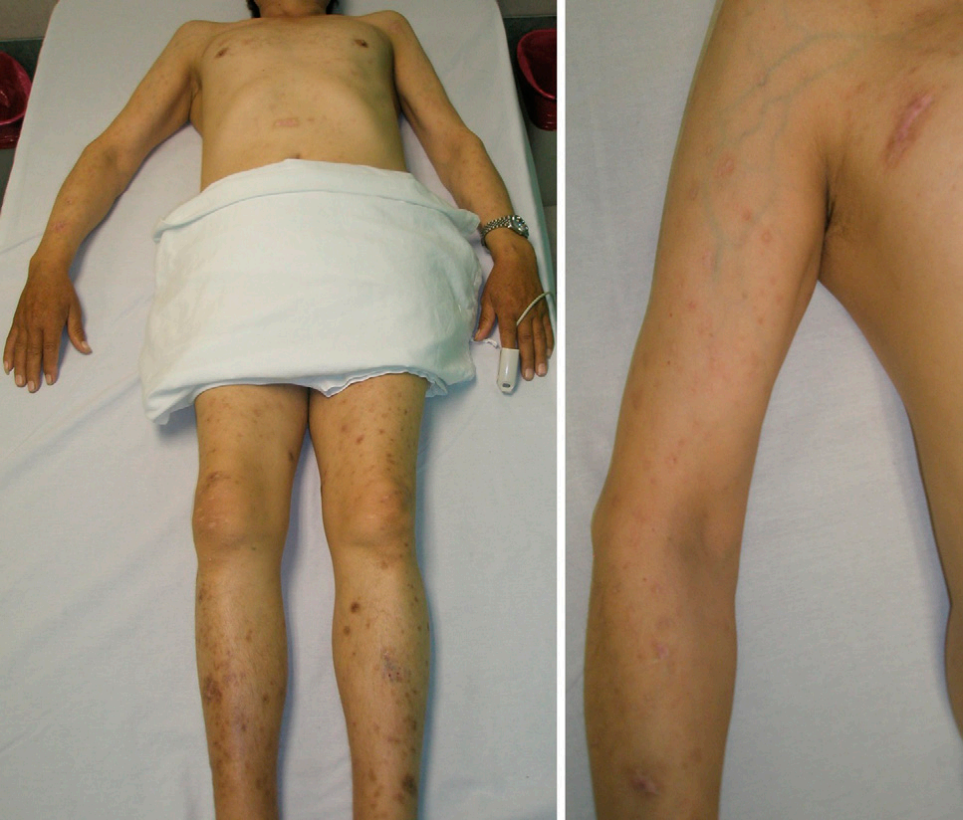
(Left) Healed lesions are observed a few months after starting treatment with itraconazole. (Right) Skin lesions were replaced by scars and some hyperpigmented spots.

).

Histoplasmosis is one of the most common systemic mycosis in Mexico, and generally, it is described in epidemic outbreaks or as an occupational illness when people accidentally inhale spores of *Histoplasma capsulatum* from soil, especially when enriched by bird or bat droppings. Chronic and progressive course is typical of disseminated histoplasmosis in non-immunocompromised adults of middle age or older, and this clinical manifestation represents 1% of all symptomatic cases.^1^,^2^ Theoretically, dissemination occurs when the fungus spreads systemically from a primary pulmonary focus during a period of months to years. High rates (27–40%) of chronic progressive disseminated histoplasmosis have been reported in some Latin American countries, especially in patients with acquired immunodeficiency syndrome (AIDS).^3^
